# Supplementary material for: Free Radical Inhibition Using a Water-Soluble Curcumin Complex, NDS27: Mechanism Study Using EPR, Chemiluminescence, and Docking
Source: Antioxidants (Basel). 2024 Jan 8;13(1):80. doi: 10.3390/antiox13010080 (PMC10812671; doi:10.3390/antiox13010080)
Supplement: Supplementary file 1 [file antioxidants-13-00080-s001.zip › antioxidants-2776892-supplementary.pdf]

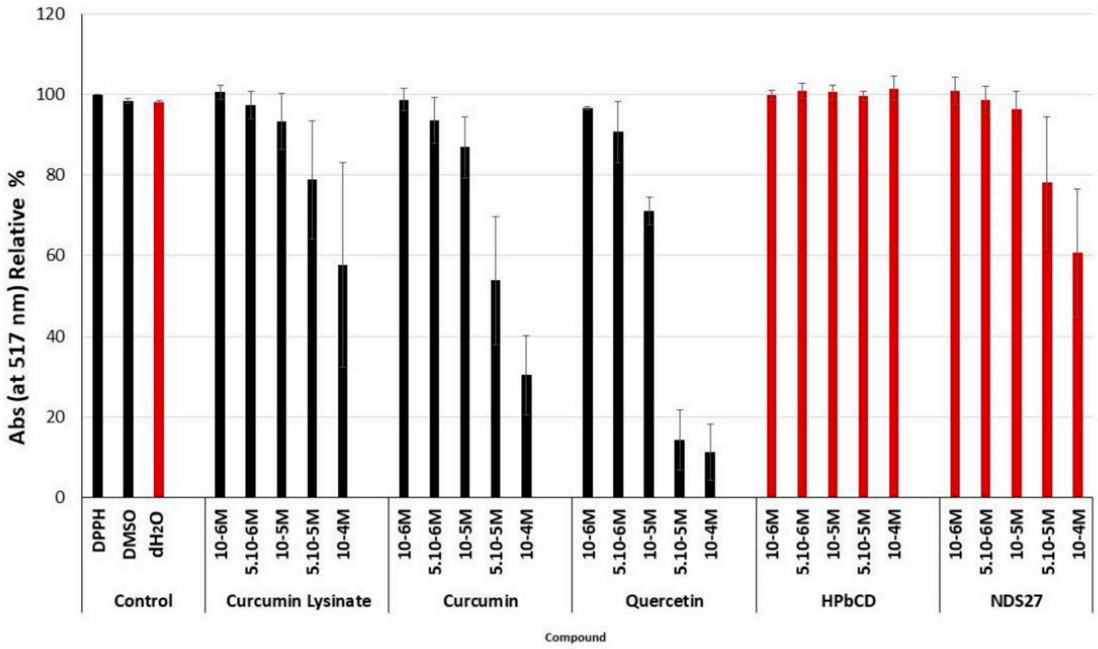

Figure S1. Effect of Curcumin, curcumin-lysine and NDS27 on the DPPH° radical by optical spectroscopy in methanol at room temperature. Quercetin was used as antiradical reference. Results are the mean ± SD of triplicate, with two independent assays (N = 2, n = 6).

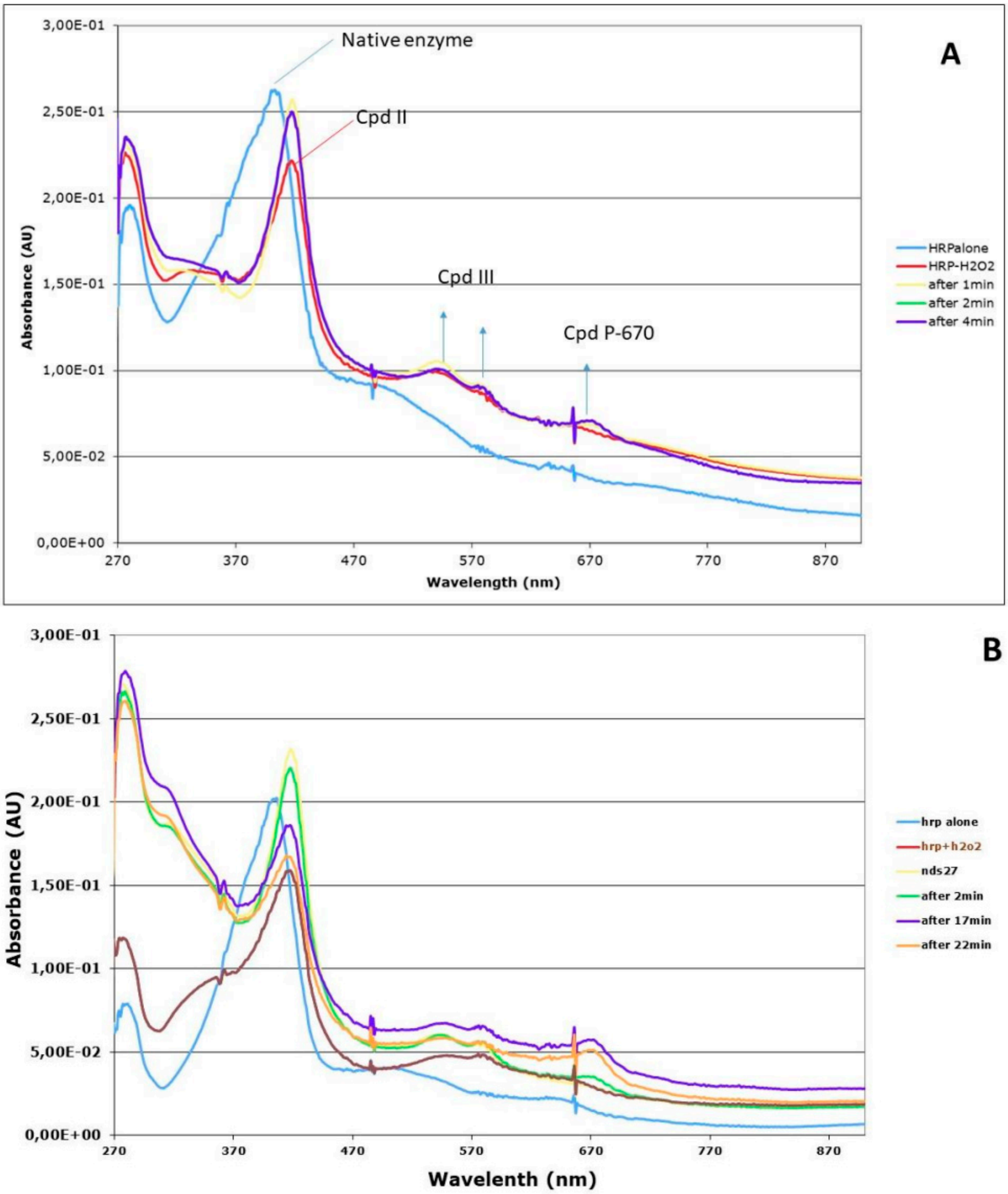

Figure S2. Optical measurements of the reactions of horseradish peroxidase (HRP) in the presence of H_2O_2 on the formation of different intermediates and the influence of NDS27 on the enzyme activity. The reaction was performed in phosphate buffer at room temperature.

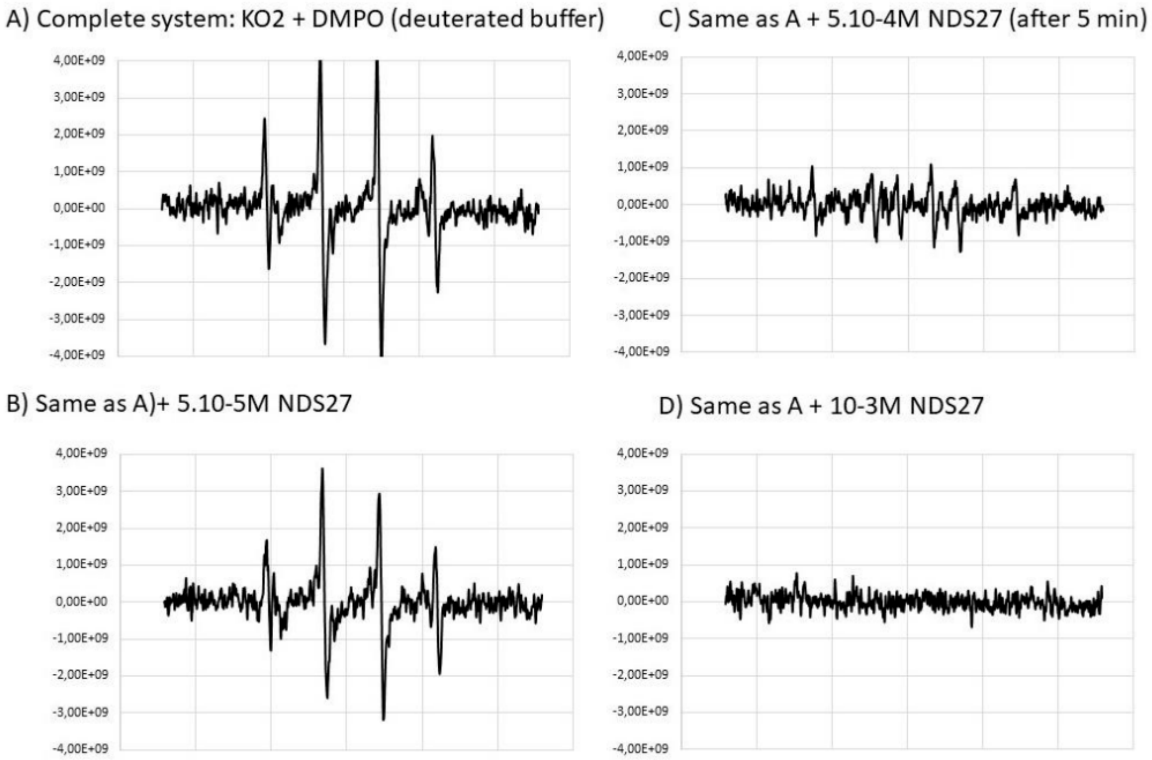

Figure S3. The EPR spectrum A of DMPO-OOH adduct obtained from the reaction of the superoxide anion radical produced by KO_2 decomposition in a mixture of deuterated water and phosphate buffer (pH 7.4). Spectra B–D are obtained in the presence of various concentrations of NDS27. DMPO was used at final concentration of 100 mM.

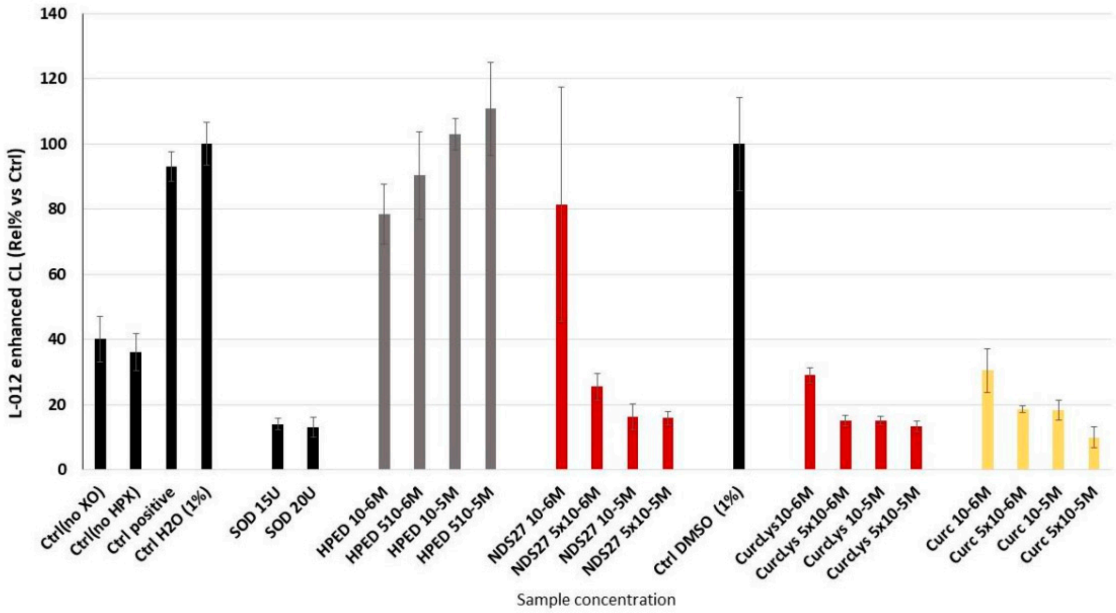

Figure S4. Effect of NDS27 on the L-012 enhanced chemiluminescence (CL) produced by superoxide anion (Hypoxanthine/Xanthine oxidase system) in phosphate buffer (pH 7.8) at room temperature. Results are mean ± SD of triplicate of two independent experiments. Controls: the complete system = Ctrl positive with hypoxanthine (HP)/xanthine oxidase (XO); Ctrl positive + 1% solvents (Ctrl H_2O and Ctrl DMSO).

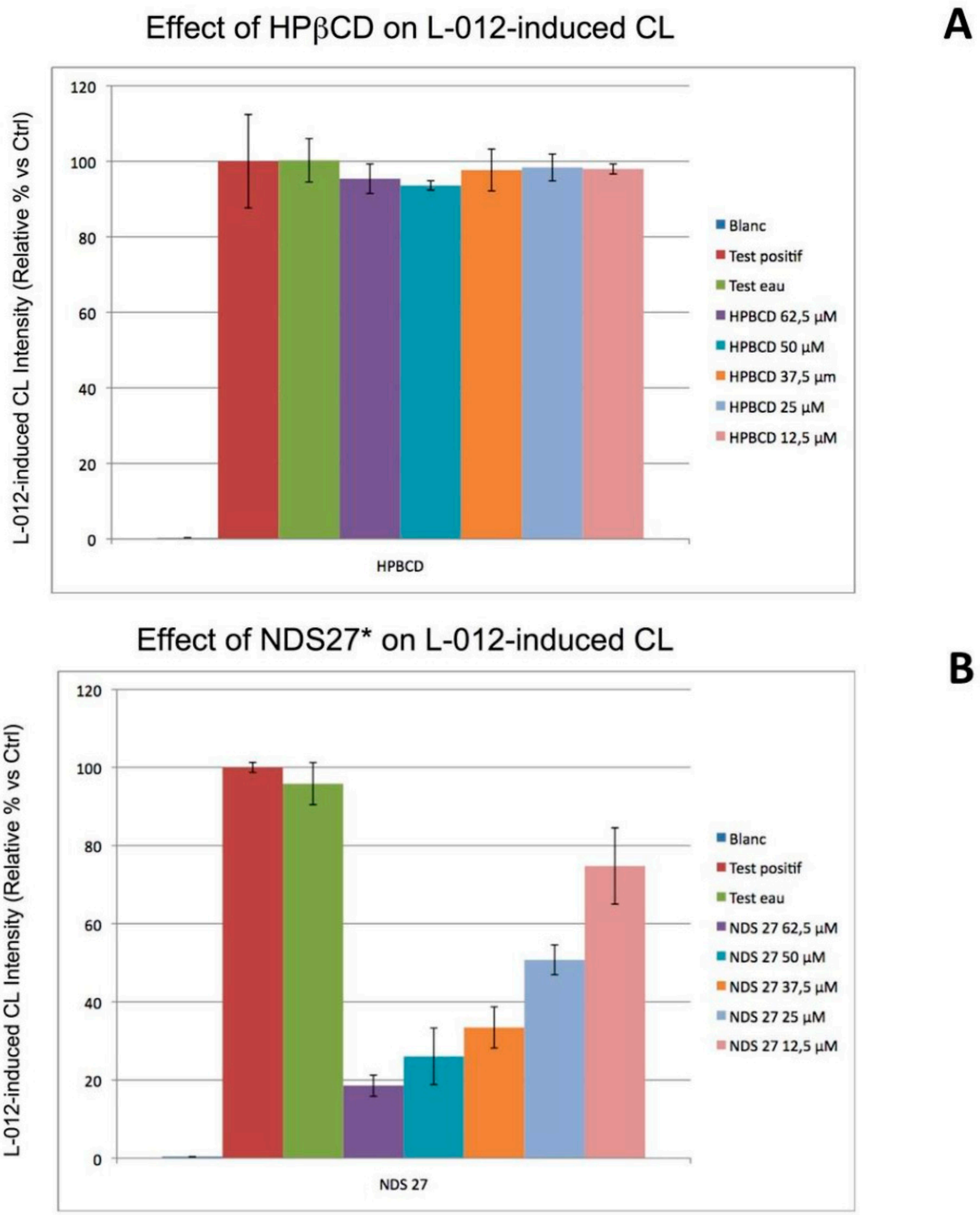

*NDS27: Lysinate Curcumin + HPβCD

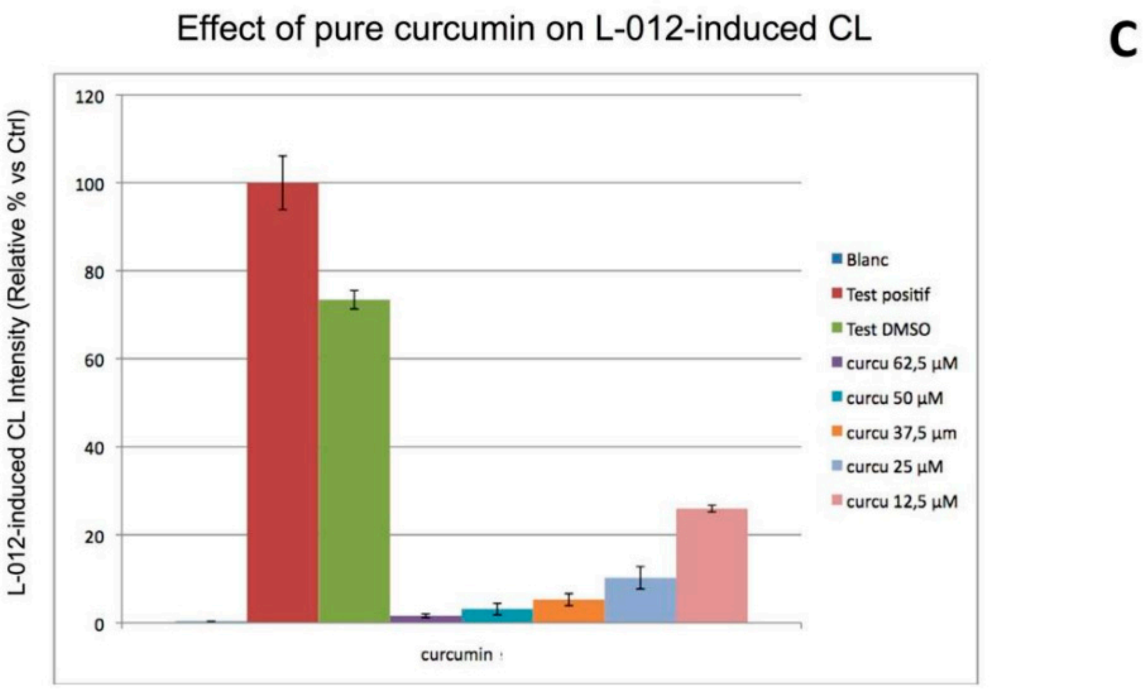

Figure S5. Effect of NDS27 compared to curcumin and curcumin-lysine on the chemiluminescence (CL) produced by the enzymatic system (HRP/H2O2) in the presence of L-012 as probe. (**A**) HPβCD, (**B**) NDS27, (**C**) Curcumin and . The results are mean +/- SD of triplicate, with two independent assays (N = 2, n = 6).

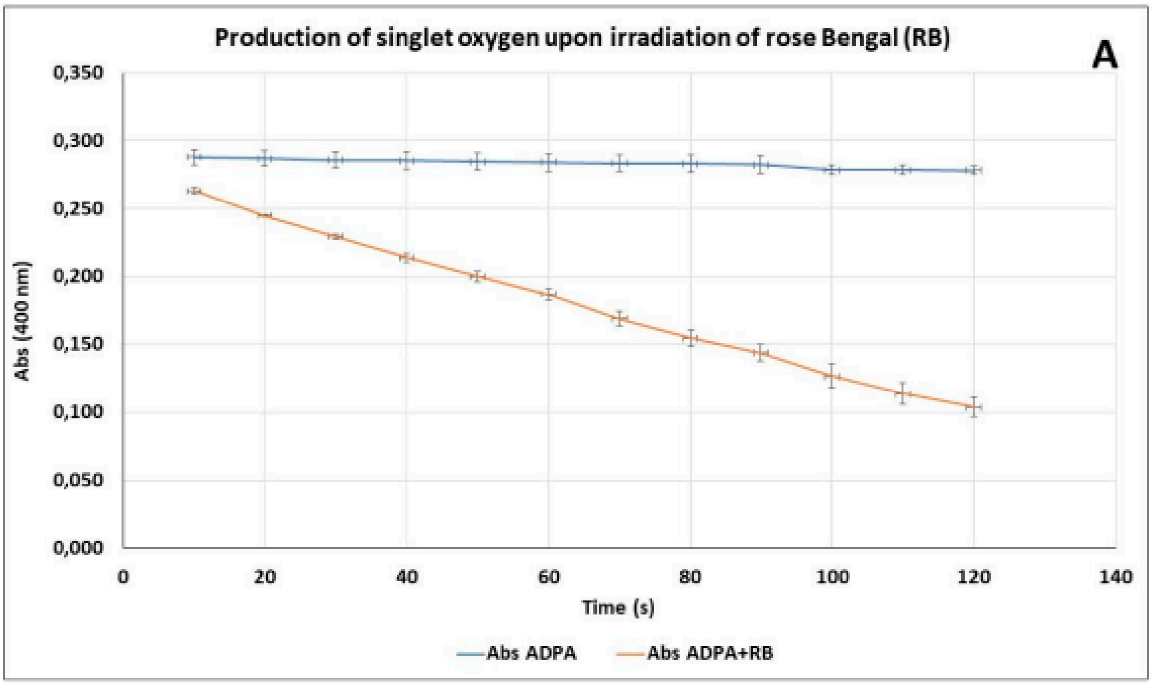
Production of singlet oxygen upon irradiation of rose Bengal (RB)
A
0,350
0,300
0,250
0,200
0,150
0,100
0,050
0,000
Abs (400 nm)
0
20
40
60
80
100
120
140
Time (s)
Abs ADPA
Abs ADPA+RB

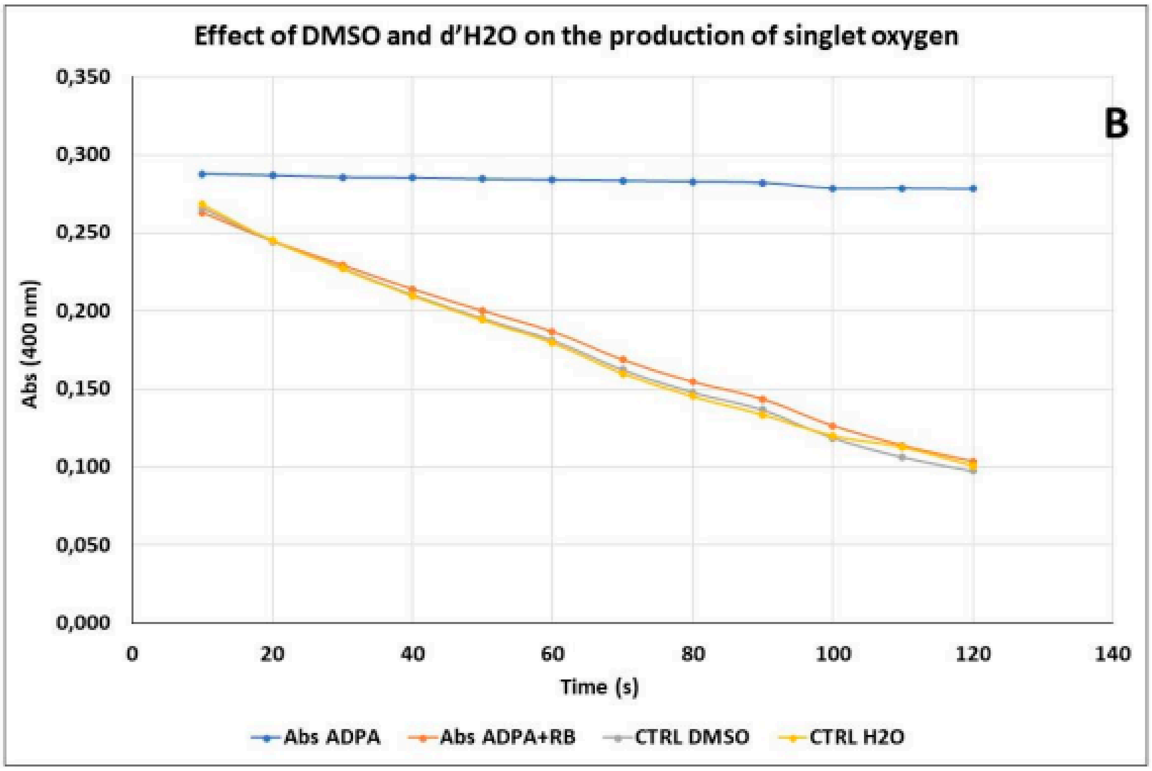
Effect of DMSO and d'H2O on the production of singlet oxygen
B
0,350
0,300
0,250
0,200
0,150
0,100
0,050
0,000
Abs (400 nm)
0
20
40
60
80
100
120
140
Time (s)
Abs ADPA
Abs ADPA+RB
CTRL DMSO
CTRL H2O

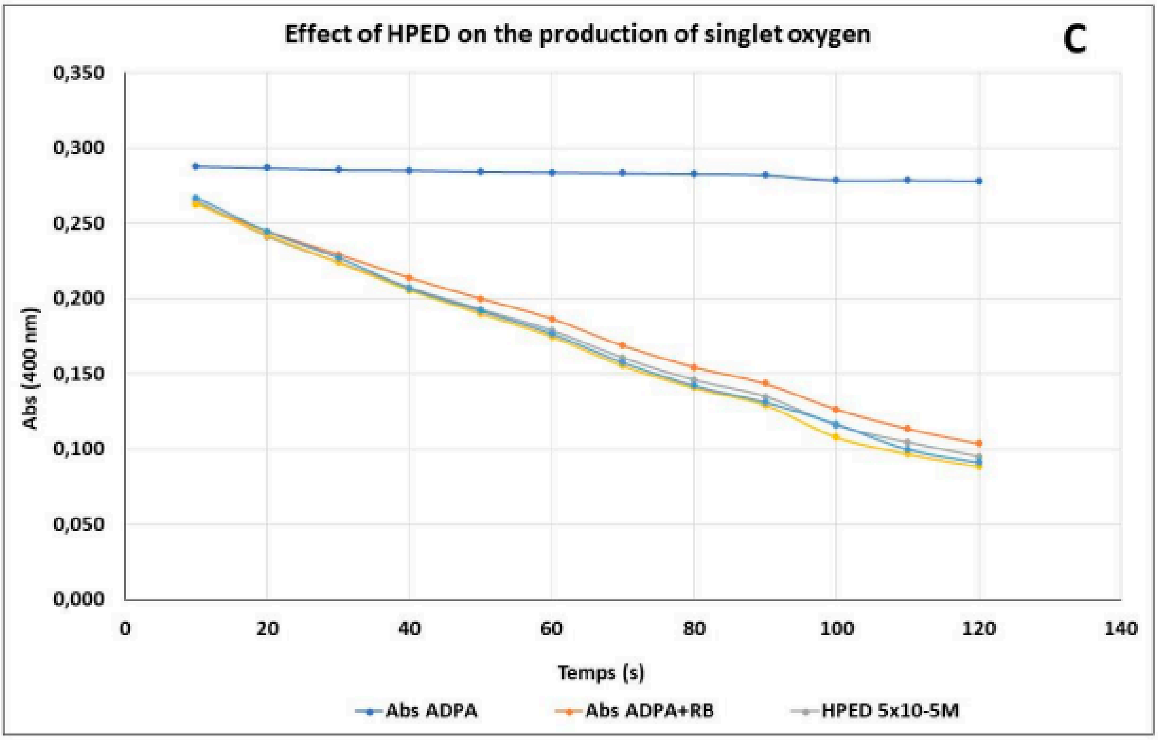
Effect of HPED on the production of singlet oxygen
C
0,350
0,300
0,250
0,200
0,150
0,100
0,050
0,000
Abs (400 nm)
0
20
40
60
80
100
120
140
Temps (s)
Abs ADPA
Abs ADPA+RB
HPED 5x10-5M

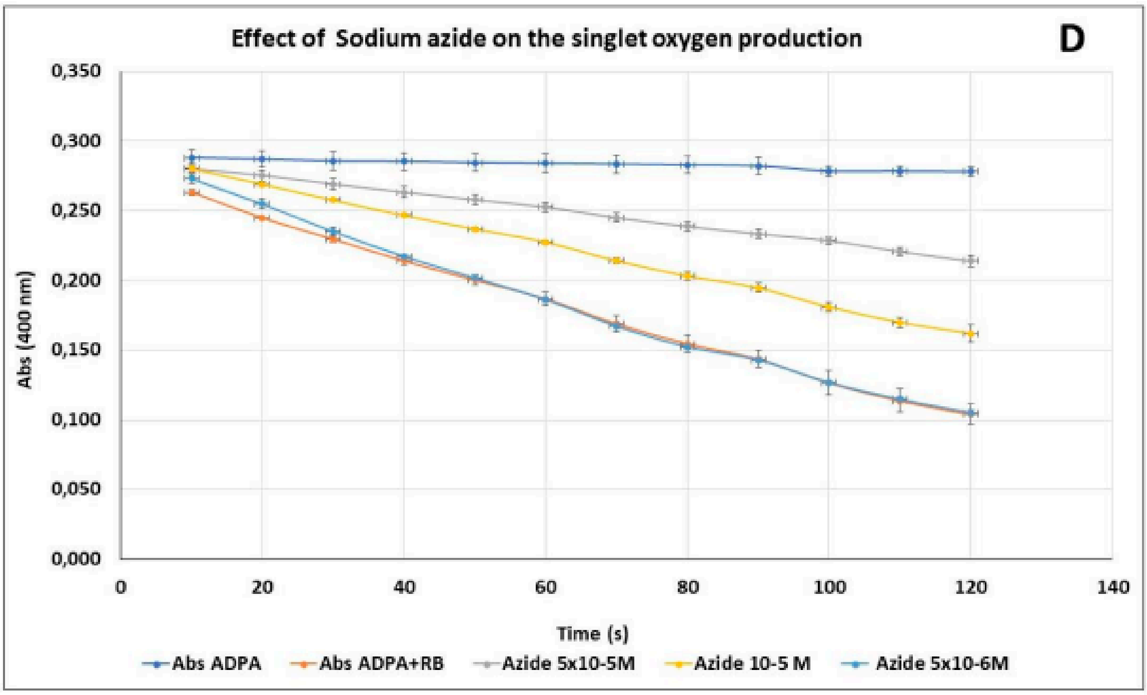
Effect of Sodium azide on the singlet oxygen production
D
0,350
0,300
0,250
0,200
0,150
0,100
0,050
0,000
Abs (400 nm)
0
20
40
60
80
100
120
140
Time (s)
Abs ADPA
Abs ADPA+RB
Azide 5x10-5M
Azide 10-5 M
Azide 5x10-6M

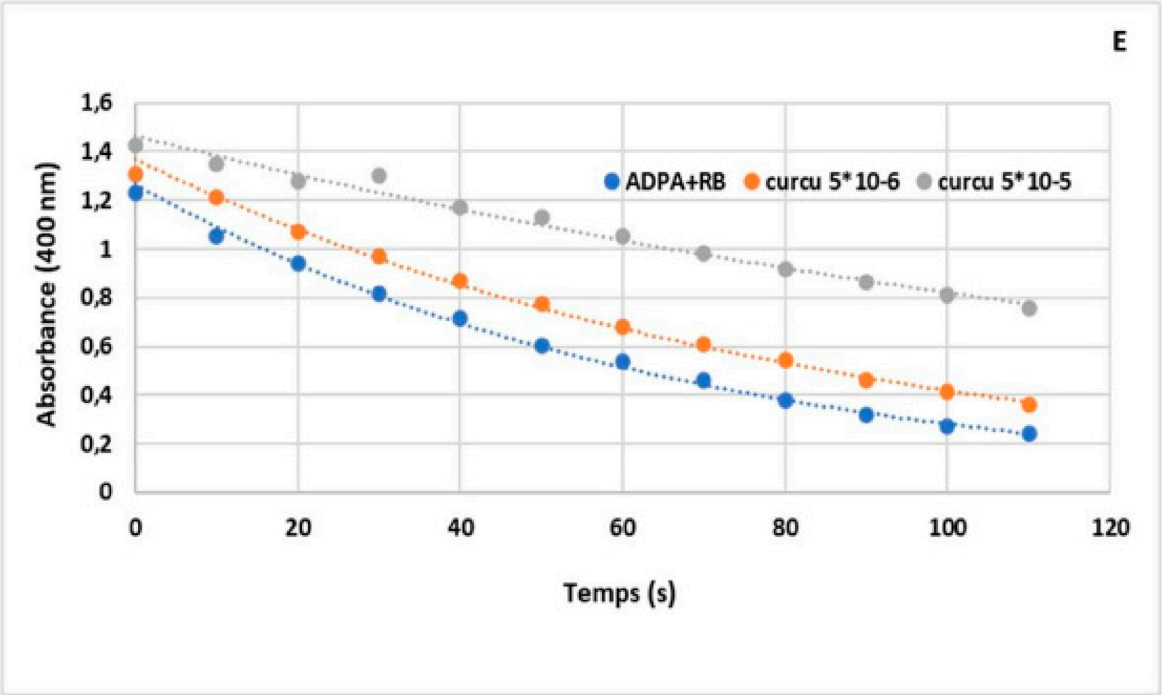

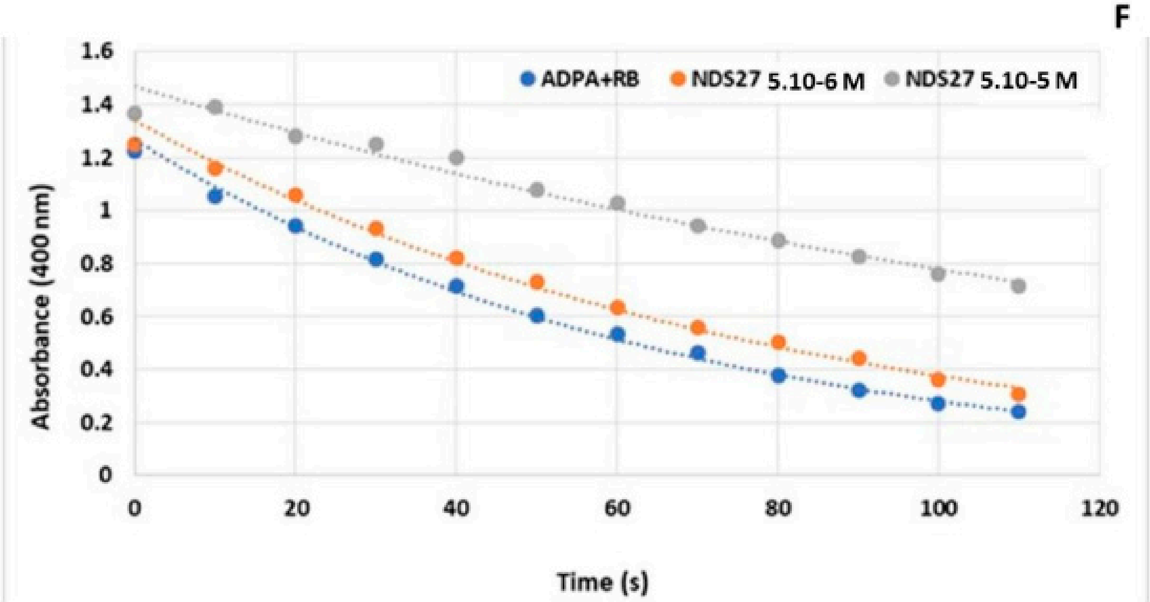

Figure S6. Optical study of the singlet oxygen quenching effect of azide (NaN3) taken as reference in comparison with solvents used water-soluble complex (NDS27) in comparison with sodium azide. The 1O2 was produced by irradiation of Rose Bengal (RB) in the presence of anthracene derivative (ADPA) in phosphate buffer pH (7.4) at room temperature. (**A**) Positive CTRL: RB + ADPA; (**B**) same as A + dH_2O and DMSO; (**C**) same as A + The excipient HPED; (**D**) Same as A + increasing concentrations of azide; (**E**) same A + curcumin ($5 \cdot 10^{-6}$ M and $5 \cdot 10^{-5}$ M); (**F**) same as A + NDS27 ($5 \cdot 10^{-6}$ M and $5 \cdot 10^{-5}$ M).

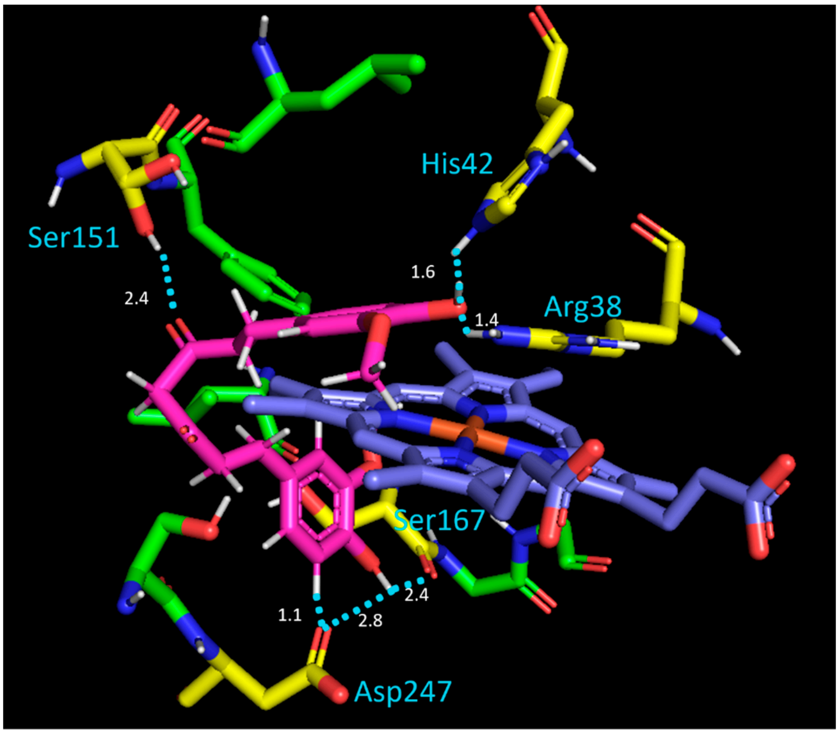

(A)

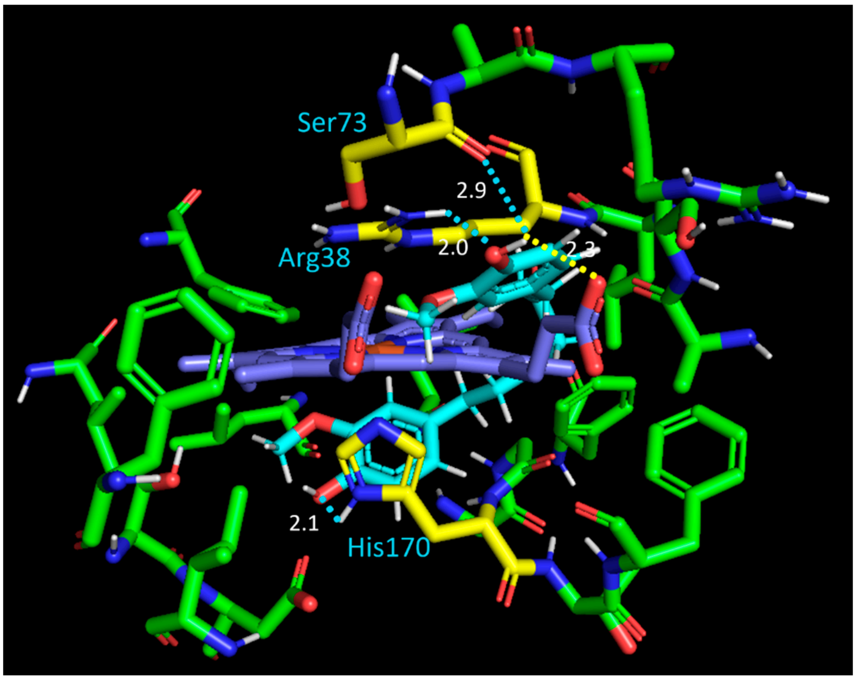

(B)

Figure S7. (**A**). Interaction of the "*E*" position of curcumin (in magenta) with residues. Interacting residues are colored in yellow, the heme in blue and the contact in dashed light blue line. The values of the interaction distance in Å are presented. (**B**). Interaction of the "*I*" position of curcumin (in cyan) with residues. Interacting residues are colored in yellow, the heme in blue, the contact in dashed light blue line and interaction with the heme in dashed yellow line. The values of the interaction distance in Å are presented.
